# Mechanistic Models of Influenza Transmission in Commercial Swine Populations: A Systematic Review

**DOI:** 10.3390/pathogens13090746

**Published:** 2024-08-31

**Authors:** Dana C. Pittman Ratterree, Sapna Chitlapilly Dass, Martial L. Ndeffo-Mbah

**Affiliations:** 1 Department of Veterinary Integrative Biosciences, School of Veterinary Medicine and Biomedical Sciences, Texas A&M University, College Station, TX 77843, USA; dana.pittman@tamu.edu; 2 Department of Animal Science, College of Agriculture and Life Sciences, Texas A&M University, College Station, TX 77843, USA

**Keywords:** influenza, swine, mechanistic model, mathematical modeling

## Abstract

Influenza in commercial swine populations leads to reduced gain in fattening pigs and reproductive issues in sows. This literature review aims to analyze the contributions of mathematical modeling in understanding influenza transmission and control among domestic swine. Twenty-two full-text research articles from seven databases were reviewed, categorized into swine-only (*n* = 13), swine–avian (*n* = 3), and swine–human models (*n* = 6). Strains of influenza models were limited to H1N1 (*n* = 7) and H3N2 (*n* = 1), with many studies generalizing the disease as influenza A. Half of the studies (*n* = 14) considered at least one control strategy, with vaccination being the primary investigated strategy. Vaccination was shown to reduce disease prevalence in single animal cohorts. With a continuous flow of new susceptible animals, such as in farrow-to-finish farms, it was shown that influenza became endemic despite vaccination strategies such as mass or batch-to-batch vaccination. Human vaccination was shown to be effective at mitigating human-to-human influenza transmission and to reduce spillover events from pigs. Current control strategies cannot stop influenza in livestock or prevent viral reassortment in swine, so mechanistic models are crucial for developing and testing new biosecurity measures to prevent future swine pandemics.

## 1. Introduction

Influenza infections of commercial swine herds cause reduced gain in fattening pigs and reproductive impact leading to substantial economic losses for the pork industry [1,2,3,4]. In addition to the economic losses, swine influenza also poses a significant threat to public health through zoonotic potential [1]. Four genera of influenza viruses have been isolated in swine: Influenza A virus (IAV), Influenza B virus (IBV), Influenza C virus (ICV), and Influenza D virus (IDV). Influenza infections in pigs are primarily caused by Influenza A virus through three major subtypes H1N1, H1N2, and H3N2 [5,6,7,8,9,10]. Influenza A is a known pathogen that is a part of the porcine respiratory disease complex, whereas Influenza B, C, and D virus infections are not known to cause respiratory diseases [11]. The primary clinical sign reported for IVB is fever and pigs infected with IVC and IVD genera are generally asymptomatic [9,12,13,14,15]. Swine influenza has significant zoonotic potential and public health implications as swine-origin viruses have been identified in humans. Some of the most noteworthy events so far were the 2009 H1N1 swine flu pandemic and the more recent spillover events of H3N2 during 2011, 2012, and 2016 agricultural fairs in the US [16,17,18,19,20].

Swine influenza A is characterized by its rapid spread among pigs, typically resulting in symptoms such as coughing, nasal discharge, fever, lethargy, and reduced appetite [21,22,23]. All three IVA subtypes have been identified as endemic on farms in numerous countries across the globe, posing substantial economic and health challenges to the swine industry [7,21,24,25]. The disease can affect pigs of all ages but is especially detrimental to young piglets and pregnant sows. Reproductive disorders associated with influenza include higher estrus rates, abortions, stillbirths, and few piglets born [2,21,26]. A cross-sectional study from Brazilian farms found influenza infection in pregnant sows is associated with a 10-fold increased risk (odds ratio: 10.93 95%CI: 1.13–105.80) of reproductive disorders such as stillbirth and fewer piglets per liter [2]. Other studies have shown that infection with influenza causes poor feed conversion ratios, requiring more feed to fatten pigs to market weight [3,27].

There are three main methods of transmission of influenza viruses in swine: direct, indirect, and aerosol transmission. Direct transmission, which generally occurs among pigs housed in the same pen, is the most common mode of disease transmission [23,28,29,30]. Infected pigs shed the virus through respiratory secretions such as nasal discharge, saliva, and mucus and pigs can encounter these secretions through nose-to-nose contact or touching contaminated skin surfaces [23,28,29,30]. Indirect transmission of influenza occurs through contact with fomites, as viruses can survive on surfaces such as feeding and watering equipment, transport vehicles, and clothing. If these surfaces are contaminated by an infected pig, the virus can be transmitted to susceptible pigs that encounter them [18,29,30,31]. Humans can spread the disease by carrying the virus on their hands, clothing, or equipment [18,30,31]. This can occur when farm workers or visitors who have been in contact with infected pigs or contaminated environments interact with susceptible pigs without proper biosecurity measures. Finally, influenza viruses can be transmitted through the air via aerosolized droplets [23,29,30,32,33]. This mode of transmission is particularly significant in densely populated swine facilities where pigs are near one another [32,33,34].

Efforts to control and prevent the spread of swine influenza include swine vaccination programs, biosecurity measures, and surveillance systems to monitor and respond to outbreaks [35,36,37]. Understanding the dynamics of influenza in pigs is crucial for mitigating its impact on animal health, the swine industry, and public health. Epidemic models are mathematical frameworks used to understand and predict the spread of infectious diseases within populations. These models can be used to analyze disease dynamics, assess the potential impact of interventions, and formulate effective strategies for disease control and prevention. There are several types of epidemic models, each with varying levels of complexity and assumptions. Epidemic models are essential tools for public health planning and response [38,39]. They help predict the course of an epidemic, estimate transmission risk, evaluate the effectiveness of interventions such as vaccination and increased biosecurity measures, and identify portions of the population most at risk for disease exposure, among others. By providing a structured approach to understanding disease dynamics, epidemic models play a crucial role in mitigating the impact of infectious diseases on populations.

In this paper, we conducted a systematic review of epidemic models for influenza transmission dynamics in domestic swine populations. We examined the modeling approaches and techniques used to develop the epidemic models and generate health outcomes. We highlighted the insights provided by these models to improve our understanding of swine influenza transmission, the effectiveness of control measures, and the risk of spillover events to humans.

## 2. Materials and Methods

The systematic review utilized PRISMA guidelines (Preferred Reporting Items for Systematic Review and Meta Analysis) [40]. Three search strings were created using terms related to pigs, influenza, and mathematical modeling (Table 1). The search string related to mathematical modeling was previously used in a systematic review [41]. The finalized search strategy linked each search string using the Boolean operator “AND”. The search strategy for each database is archived on searchRxiv; the DOI for each search is provided in the data availability section. On 17 May 2024, DCPR executed searches on PubMed, Web of Science, CABI, EMBASE (OVID), MathSciNet, Academic Search Ultimate, and Medline (Ultimate). A ‘snowball’ search was conducted to locate other modeling studies by scanning the reference lists of studies eligible for full-text review. One researcher (DCPR) independently performed screening and full-text review using Covidence systematic review software (June 2022 release), Veritas Health Innovation, Melbourne, Australia. Covidence is a web-based collaboration software platform that streamlines the production of systematic and other literature reviews.

To be included, a model had to explicitly account for influenza transmission dynamics in domestic swine *Sus scrofa domesticus*. Studies were excluded from the final extraction if the focus was on a species other than *Sus scrofa domesticus*, such as wild boar. Mathematical models that were non-population-level, virological, or genomic were excluded. Additionally, studies not classified as original research or not published in English were excluded.

A table shell was generated in Google Sheets and used by one author (DCPR) to extract data from eligible studies. Accuracy of extracted data was verified by MLNM; disagreements on extraction entries were resolved by discussion. Studies were grouped by the interactions between species; therefore, models are categorized as swine only, swine–avian, or swine–human. For the models containing all three species those were classified as swine–avian. Information about the virus such as specific strains of influenza, transmission type, and control strategies were extracted. Contextual details of the model like geographic location and type of farm were extracted to better understand applicability of models to specific scenarios. In this paper the terms herd and farm are used interchangeably.

## 3. Results

**Figure 1:**
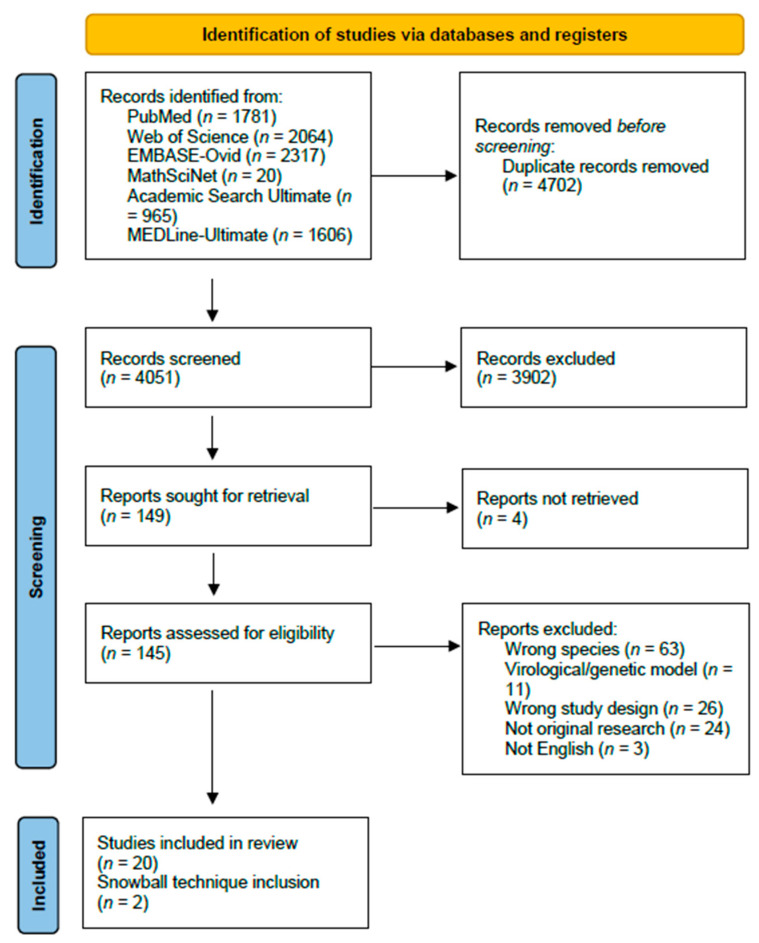
PRISMA 2020 flow-chart of article identification, screening, eligibility, and inclusion.

The search query generated 8734 studies, of which 4683 were duplicates. A total of 4051 studies were screened and 149 were assessed for eligibility through full-text readings. When seeking papers for retrieval, four papers were not available online. From the search, a total of twenty-two papers were identified as mechanistic models of influenza transmission in domestic swine populations, and two papers were identified through references (Figure 1). These studies were published between 2005 and 2023, with most of the studies published between 2013 and 2019 focusing on data from the 2009 swine influenza pandemic.

### 3.1. Model Methodologies Overview

Three classes of mechanistic models were reviewed: deterministic compartmental models (*n* = 7), stochastic compartmental models (*n* = 15), and an agent-based model (*n* = 1) (Table 2 and Table 3). The agent-based model was constructed and simulated using the North American animal disease spread model (NAADSM) [42]. NAADSM 3.1.24 is an epidemic simulation software developed by Harvey et al. and used for assessing the spread and control of disease between livestock herds through microsimulation models [43]. This was the first instance of humans being incorporated in NAADSM [42]. This agent-based model tracks units such as herds and households with count and spatial distribution fitted to Ontario, Canada [42]. Transmission for this model could occur between farms by movement of contaminated fomites or individuals (indirect), and movement of swine between farms [42]. Parameter settings were enabled in NAADSM to allow for airborne and direct transmission meant to simulate pathogen spread between adjacent pens/farms through fences or inconsistencies in biosecurity measures [42]. The remaining models were compartmental models where individuals were classified according to disease status such as susceptible, infected, exposed, and recovered (Table 2). These compartmental models were both deterministic (fixed output) and stochastic (probability-based output) in nature. Most studies modeled swine influenza transmission within a single farm (*n* = 18), with the rest focusing on between-herd transmission (*n* = 3) and country-level transmission (*n* = 1).

Compartmental models use the first letter of the compartment’s name as an abbreviation to distinguish the health state of the individuals and show the progression of the disease. Commonly, compartmental models are structured with susceptible, *S*, infected, *I*, recovered, *R*, and in some models an exposed, *E*, class (Table 2). Four models added an *M* compartment for animals with maternally derived antibodies (MDAs) that grant the animals passive immunity [45,46,47,51] (Table 2). Another commonly added compartment was *V* for a vaccinated class (Table 2). For example, White et al. modeled vaccination as a series of compartments with reduced transmission rates relative to unvaccinated [55]. Cador et al. consider vaccination through the simple addition of a class of animals whose parameters related to transmission are smaller and have the added assumption that these animals can experience, at most, one infection [47]. To incorporate the co-infection of viruses, Cador et al. added a *Y* compartment that allows for the animals to be shedding both subtypes at the same time [47]. Coburn et al. expanded their SIS model to include a *J* compartment for super strain infections in human and swine species; for pigs, this enables them to transmit infection to humans [57]. Super strains are defined as genetic recombinants of influenza from the swine population and are highly virulent to humans [57]. This model assumes pigs can be infected by either humans or birds and can produce a super-strain, but humans and birds do not infect each other [57]. Chen and Cui also added a special infected compartment to pigs, *X*, for a variant virus-infected class that could infect humans [56].

Model fitting to epidemiological data was conducted in five out of twenty-two studies. Methods included Bayesian inference [4,45], least-square optimization [57], generalized linear modeling [44], and successive iterations of the differential equations model [17] (Table 2). Additionally, two models [4,51] conducted model validation using ground truth (Table 2). Ground truth represents information acquired by direct observation. Here, models’ outputs were compared with empirical data about the outbreak of interest. The key difference between model fitting and model validation is that the data used to validate the model is not used in model fitting to generate the model’s parameters. Sensitivity analyses were undertaken in eight of the nineteen studies: researchers used methods such as univariate analysis (*n* = 6), scenario analysis (*n* = 1), Pearson’s partial rank correlation coefficient (n = 1), and Latin hypercube sampling (*n* = 1) (Table 2).

Univariate sensitivity analysis was used to assess the influence of transmission, movement, and birth/death rate parameters on disease prevalence in swine populations [48,50,51,52,61,62] (Table 2). For example, Widayanti et al. found that pig mobility, the chance of success of pigs becoming infected, and the coefficient of periodic transmission had a positive relationship with the basic reproductive number (R_0_), and pig death rate had a negative relationship with R_0_ [62]. Etbaigha et al. tested the robustness of their model’s predictions for a range of values for three model parameters related to direct and indirect transmission rates among pigs in a farrow-to-finish farm. They showed that their results were not sensitive to these values [48]. Using a univariate analysis, Reynolds et al. showed their result was robust to changes in the size of the farm [52]. Pitzer et al. showed that there is a negative relationship between the length of the farrowing interval and disease persistence in each farm, and the positive relationship between farrowing interval and model-predicted variance for seroprevalence also increased [51]. Saenz et al. developed a multispecies influenza transmission model, including pigs, workers, and individuals in the local community [61]. They calculated R_0_ in each species to estimate the transmission potential of the pathogen. Using a univariate sensitivity analysis, they showed that as R_0_ for swine becomes larger with respect to R_0_ for humans, the swine became more important in the epidemic dynamics for humans [61]. Moreover, they showed that doubling the contact rate between infected workers with swine would decrease the length of the epidemic and the epidemic peak will occur 20 days sooner [61]. To model a country-level spread of a swine influenza virus, Nelson et al. developed a coupling matrix to represent the flow of swine between countries; this matrix consisted of a fixed value referred to as free parameter c [50]. Nelson et al. conducted a sensitivity analysis by testing a range of values for R_0_ and the free parameter. They showed that changes in the values of these parameters would affect the synchronicity of epidemics in various locations and the time course for a global epidemic/pandemic [50].

Wong et al. developed a model to investigate H3N2 transmission among humans who attended a 2011 agricultural county fair in Pennsylvania [17]. They performed a scenario analysis, by considering a scenario where 75% of the cases of H3N2 infections among humans came from contact with infected pigs at an agricultural fair [17]. Under this assumption, the probability of a human contracting the infection per minute of contact with pigs reduced to 0.017 from 0.025, obtained under the assumption that all cases of H3N2 infections occurred at the fair [17]. The final methods of sensitivity analysis identified in this review are Pearson’s partial rank correlation and Latin hypercube sampling. These were employed to evaluate the relative influence of each parameter on the model’s predictions. White et al. specifically used Pearson’s partial rank correlation to identify links between parameters and outputs after the effect of the remaining model parameters was accounted for [55]. Positive correlation with endemic prevalence was observed with early weaning, the direct transmission rate of piglets with maternal immunity, and the loss of immunity rates in sows and gilts [55].

Three models performed an uncertainty analysis of model parameters (Table 2). Probabilistic uncertainty analysis involves testing a range of parameters over a confidence interval or across a probability distribution. It is often used when a parameter value is unknown or has high levels of uncertainty. Reynolds et al. analyzed the outputs of their model over a range of transmission rate values across the 95% confidence interval, finding the results to be robust to this interval [52]. Another method was used by Kontwowicz et al., where a distribution of values over 5000 Monte Carlo samples were taken for each model parameter to understand the epistatic uncertainty of the model [60]. To save on computational power, the parameters generated by the simulations were used in a deterministic model and the outputs of these models were compared with that of the stochastic model [60]. Cador et al. used a similar technique to Kontowicz et al. but used the outputs of their uncertainty analysis to set the values of the between-batch airborne transmission rate, the post-infectious immunity period duration, and the immune protection efficacy after a previous challenge acting on the duration of the active immunity period [46,60]. The researcher chose their parameters based on the parameter values with the most stable outputs of the stochastic model [46].

#### 3.1.1. Metapopulations

Two types of metapopulation models were implemented by these studies for population dynamics (Table 2). These metapopulation models modulate infection rates by age group and the movement of swine between locations. Models looking at the farrow-to-finish outfits implemented metapopulation models to recreate the movement of swine within the farm. The high turnovers of animals in farrow-to-finish operations require intricate metapopulation models to accurately describe the movement of animals within facilities. Two sub-populations were incorporated into the model breeding units and growing units: this mimics the physical separation of these two populations and the constant flow of piglets from the breeding unit to the growing unit [46,47,51,52,55,60] (Table 2). Time spent in each unit was parameterized based on current agricultural practices on the development and gain of pigs [46,47,51,52,55,60]. A different version of a metapopulation model was implemented by Nelson et al. for moving pigs between countries along trade routes to identify areas where the virus may reassort [50]. Geographic regions were divided into 146 patches based on the trade network data [50]. To track the progress of the epidemic, a spatially extended chain binomial system which linked daily incidence and the number of newly recovered in each patch was developed [50].

#### 3.1.2. Parameterization

Only 8 of the 22 studies calibrated their model to empirical data [4,17,44,45,50,51,53,57] (Table 2). Three models were parameterized using laboratory experiments data to quantify the transmission of H1N1. Allerson et al. and Andraud et al. sought to quantify contact transmission and airborne transmission in the presence of MDA using data from sows and their piglets vaccinated at different time points [44,45]. Romagosa et al. used data on piglet vaccination using heterologous and homologous vaccines and calculated transmission from contacts data between infected and susceptible pigs [53]. Coburn et al. used two sets of empirical data to parameterize the swine, avian, and human populations [57]. The human parameters were fitted to data from the avian seasonal H5N1 strain from 2005 using a logistic equation [57]. It must be noted that this model assumes the human and avian populations to have the same parameter values for the density of susceptibles, intrinsic growth, and carrying capacity [57]. The swine parameters were calibrated to data from the Food and Agriculture Organization (FAO) of the United Nations, and the remaining unknown values were assumed to be like the human parameters [57]. Nelson et al. also used FAO data to parameterize the trade networks for between-country transmission from 1969 to 2010 and combined this data with whole-genome sequences from swine between 1960 and 2013 [50]. Er fit his model to seroprevalence data using a Bayesian Inference approach [4]. These seroprevalence data were collected yearly from roughly 500 Norwegian herds, testing about 10 pigs per breeding herd and 50 pigs per fattening herd. Infection in herds was confirmed by enzyme-linked immunosorbent assay (ELSIA) and hemagglutination tests that found H1N1pdm09 was the only influenza A virus in the Norwegian herds [4]. Wong et al. parameterized their model using empirical data from a Pennsylvania county fair. The data were collected through a retrospective cohort study of agricultural club members who attended a fair [17]. A survey was used to collect data on the number of infected swine, the duration of contact with swine, the number of humans in contact with swine, and other related parameters [17].

### 3.2. Epidemiological Characteristics

Studies were categorized by the populations involved in the model. Out of the nineteen mechanistic modeling studies, ten considered swine populations only, three incorporated an avian population, and six models added a human population (Table 3). In broad terms, the objectives of the models often involved some combination of understanding outbreak dynamics, assessing viral recombination dynamics, forecasting, evaluating control strategies, or analyzing transmission determinants. Most models did not specify the strain of influenza used to parameterize the model (*n* = 8) or mentioned influenza A without providing strain information (*n* = 6). Among the models that did provide virus information two subtypes were identified: H1N1, the most common (*n* = 4), and H3N2v (*n* = 1) (Table 3). Six models tailored model parameters to specific geographic locations: Ontario, Canada (*n* = 2); Thailand; the Netherlands; Lerma, Mexico; and Norway (Table 3). The transmission setting used most often by the models was the farrow-to-finish farm (*n* = 6), though other farm structures were observed, like growing unit, fattening unit, and an outfit with both swine and poultry.

#### 3.2.1. Control Strategies

Control strategies were an important factor considered by 14 of the modeling studies. Twelve of the fourteen studies employed a vaccination strategy, and six studies implemented a non-pharmaceutical intervention (Table 3). Vaccination was applied to both human and swine populations. When vaccination was performed in the human population, this took the form of assuming that a fixed percentage of the population were fully immune [42,61,62]. There are numerous vaccination strategies for influenza in swine; the two common practices are batch-to-batch and mass vaccination. The vaccination of different age groups was also considered, such as gilt, pregnant sow, growing pigs, and piglets after birth vaccination [44,45,47,48,53,55] (Table 3). Additionally, different types of vaccination were considered: heterologous and homologous vaccines [44,52,53,55]. Homologous vaccines are prepared using an isolation of the strain affecting the herd and heterologous vaccines are prepared with a strain from a different herd and may elicit cross-protection [44,52,53,55]. Non-pharmaceutical interventions are defined as disease control strategies that do not involve medication or vaccination. The isolation of infected pigs, the concurrent export of weaning pig batches, the separation of gilts by placing them into a development unit, all-in-all-out herd management, and the varied timing of gilt introductions to the breeding herd are non-pharmaceutical interventions that have been modeled [47,51,55,60]. For the human population, non-pharmaceutical interventions included changing worker routine and improved personal protective equipment with masking [60].

#### 3.2.2. Swine-Only Models

The swine-only models often structured the population to mimic the environment of a farrow-to-finish farm (Table 3). These models are heavily connected, and their origin can be traced back to a single epidemic model published in 2014 by Reynolds et al. [52]. This model was developed to analyze the dynamics of swine influenza infection dynamics at the farm level. They used a metapopulation model that divides swine by age into different sections of the farm and moves them through each stage of production: gilt unit, breeding/gestation, farrowing, and weaning [52]. They used their model to evaluate the effectiveness of vaccination using a homologous vaccine (vaccine prepared with the isolate recovered from the specific population in which it will be used) or a heterologous vaccine (vaccine prepared with isolates distinct from the specific strain in the population). Reynolds et al. found that the breeding section of a farm experiences higher disease prevalence than a wean-to-finish farm and attributed this result to the constant introduction of new susceptibles into this population [52]. They showed that homologous vaccination during the pre-farrow period delays the outbreak, lowers the number of infectious pigs at the peak by a third, and increases the number of infectious gilts and sows after the peak compared with no vaccination [52]. Additionally, they showed that vaccination does not significantly reduce the number of infectious piglets [52]. However, in the wean-to-finish portion of the farm, they showed that vaccination with a homologous vaccine eliminates the virus from the populations, but a heterologous vaccine has little effect on the number of infectious pigs [52]. This model was limited to a single influenza strain and assumed a fully naive population. This may have led to an overestimation of infected animals and the potential impact of vaccination [52].

Cador et al., Andraud et al., and Pitzer et al. expanded the Reynolds et al. model to analyze the impact of maternally derived antibodies (MDAs) on influenza A virus (IAV) transmission in a swine farm [45,46,51,52]. They showed that MDAs are a leading determinant of transmission and the persistence of IAV, especially in the farrow-to-finish farm models [45,46,47,51,52]. Cador et al. showed that when more than two-thirds of piglets possessed MDAs, fewer animals would be infectious at the peak of an outbreak, but the duration of the epidemic would be longer [46]. Pitzer et al. expanded the analysis by investigating the impact of herd size on disease persistence in the presence of MDAs. They identified a critical herd size of 3000 animals needed for IAV transmission to persist in swine farm operation; this equates to a large-scale operation [51]. Andraud et al. used a laboratory transmission experiment and a mathematical model to evaluate the impact of single dose vaccination on disease transmission in weaned piglets in the presence of MDA [45]. They showed that when sows were vaccinated, resulting in MDAs in piglets, the likelihood of the virus persisting in a farm ranged from 33% to 67% five years after introduction [45]. Conversely, in cases where piglets did not have MDA, the virus periodically went extinct after four years post-introduction [45]. In considering these results, one should keep in mind the fact that the laboratory transmission experiments might not accurately reflect transmission dynamics in a real-life production setting, and that this model only considered the dynamics of a single circulating influenza strain.

A latter model by Cador et al. expanded their previous analysis to evaluate the risk of the co-circulation of influenza viruses after a reassortment event leading to the emergence of a new virus and assess the ability for concurrent batch export and vaccination to lead to the viral elimination in this farm structure [47]. The researchers could not locate quantitative data pertaining to the amount of virus shed by vaccinated animals, so these parameters are assumed in the model [47]. The model’s analysis of two co-circulating influenza subtype viruses showed three co-circulation patterns: (a) subtype *i* infection closely followed by subtype *j* (or vice versa), allowing brief co-infections; (b) simultaneous infections by both subtypes, resulting in moderate separate infections but many co-infections; and (c) subtype *i* infections in young piglets, followed by subtype *j* in finishing rooms months later, causing two distinct outbreaks [47]. Between the two control methods, the export of piglet batches was shown to be the most effective measure due to the synergistic effect of disrupting infection through consecutive exports [47]. Vaccination could not produce virus fade-out in the model but was deemed beneficial at reducing persistence in the breeding sow sub-population [47]. Etbaigha et al. investigated the effectiveness of vaccination and reduced direct contact in a farm setting, which allowed for reinfection, on IAV transmission [48]. They showed that neither of the control strategies could eliminate the virus from the farm [48]. Specifically, they showed that neither pre-farrow vaccination nor vaccination at the end of the first week led to a reduction in the number of infected piglets [48]. In the case of minimizing indirect contact, it was shown to delay the start of disease outbreak in the farm but had no impact on the number of infected [48]. It must be noted that no empirical data were used to parameterize vaccine immunity and indirect transmission rates in this model [48]. White et al. evaluated a wide range of control measures including vaccination (homologous and heterologous vaccine), weaning timing, and gilt separation, among others [55]. They tested fourteen different disease control strategies independently and in combination to explore their impact on the transmission dynamics of H1N1 [55]. The most effective control strategy was mass vaccination with a homologous vaccine every two months, as this reduced endemic prevalence and the probability of infection [55].

Allerson et al. and Romagosa et al. conducted laboratory experiments and used their data together with a mathematical model to quantify the transmission of H1N1 in piglets and assess the effectiveness of vaccination on disease transmission dynamics [44,53]. The results of the transmission experiments were used to parameterize simple SIR models for disease transmission within pens. In a fully naive population, Romagosa et al. saw the entire pen becoming infected in 84% of the simulations without vaccination [53]. Simulations under the use of heterologous vaccines resulted in 7% of the entire pen becoming infected and 40% with no new cases [53]. Allerson et al. expanded Romagosa et al.’s study by incorporating MDA in transmission dynamics and obtained similar results [44,53]. Eighty-nine percent of the simulations with no vaccination resulted in all pigs in the pen becoming infected [44]. Vaccination with heterologous and homologous vaccines resulted in 80% and 0.3% of the simulations having all the pigs become infected [44]. It should be noted that parameters from these experiments come from highly controlled environments, not a production setting, and the protection from vaccination may be an overestimate [44,53].

Toft et al. developed their model to optimize the decision-making process regarding treatment and vaccination control strategies for a fattening unit to maximize production [54]. This model considered one section of animals despite production facilities having multiple sections that have the potential to infect each other [54]. Additionally, this model assumes the number of infectious pigs is known and infected animals have no incubation period [54]. When comparing treatment and vaccination, treatment was 40% less effective at reducing the contact rate of infection meaning pigs were more likely to come into contact with the infection [54]. Additionally, the researchers concluded that treating and vaccinating the pigs was redundant and an unnecessary cost [54]. But the authors acknowledge vaccination is expensive and unprofitable, as the cost associated with vaccinating animals reduced profit margins, in numerous scenarios they tested [54]. They deemed both treatment and vaccination as unreasonable control methods for influenza in fattening units due to treatment being infective and vaccination being unprofitable [54].

Three papers considered a different transmission unit to the farm level. Er and Mateus-Anzola et al. developed models where the basic epidemiological unit was herd and Nelson et al. investigated transmission between countries [4,49,50]. Er sought to forecast yearly influenza seroprevalence in Norwegian pig populations [4]. Er modeled between-herd transmission in Norway parameterized using the 2009–2010 data [4]. A disadvantage of using serosurveillance is it detects antibodies rather than active virus so positive herds may not indicate new infections from an active virus [4]. This is especially true for pig herds that tested positive in consecutive years. When model outputs were compared with longitudinal seroprevalence data from 2010 through 2020, they found a good fit from the 2010 to 2016 data but an average overestimation of 17.25% for 2017 through 2020 [4]. The model did not track well for the data after 2016 because of changes in the force of infection [4]. This model can be considered an oversimplified model because it is an aggregated representation of all farm types in pig production even though farms with farrowing had higher seroprevalence compared with fattening units [4]. The Mateus-Anzola et al. model describes the transmission between backyard farms in Lerma, Mexico [49]. Specifically, the researchers investigated how the interconnectedness of trade networks leads to the rapid infection of farms across the region [49]. The connectivity level of the farms was shown to be a critical factor in disease spread. For example, with high farm connectivity, it took as few as 5 days for half the farms simulated in the model to become infected and persist [49]. The researchers acknowledged that their model was constructed using outdated national census information and limited data about the movement between backyard farms and markets, so this must be considered when analyzing the models’ outputs and applicability [49].

On the global scale, Nelson et al. developed a meta-population model for trade networks to forecast geographic locations where viral reassortment is likely [50]. Using China, France, Canada, Mexico, and the United States as starting points, predictions of the simulations confirmed the phylogenetic results that the long distance transmission of influenza continuously happens along trade routes [50]. This model did not account for within-country dynamics or the likelihood of initial viral emergence within seed countries. The analysis also showed the continuous long-distance transmission of influenza along trade networks as early as the 1970s [50]. Nelson et al. concluded more surveillance efforts are needed in Latin America and Asia as the United States and China produce the bulk of data and are not representative of virus diversity [50].

#### 3.2.3. Swine–Avian Models

The primary objective of the swine–avian models can be summarized as analyzing multi-species dynamics (Table 3). Two studies included swine, avian, and human populations in their models [56,57]. Chen and Cui developed a cross-species model to analyze influenza transmission between the different species [56]. Chen and Cui found that the per capita incidence rate from birds to pigs did not significantly impact the human epidemic [56]. The results of the model indicate that pigs, the intermediate host, play a more significant role in human epidemics than birds, the natural reservoir [56]. This model provided little information about infectious contacts between the different host species. Additionally, significant uncertainty in the model outputs were observed, as the model was parameterized to a generic setting. Coburn et al. developed a theoretical model to investigate the epidemiological outcomes when pigs function as a mixing vessel for a reassorted “super-strain” virus from three species: pig, human, avian [57]. Coburn et al. showed that super-strain infections from swine to humans often produced the highest mortality for humans during the initial outbreak, and cross-species interaction could lead to the continuous introduction of super-strains into the human population [57]. The model was used to investigate epidemic dynamics over centuries but did not account for the possibility that virus reassortment could change disease transmission patterns between the different species. The third study by Zhuang et al. developed a swine-poultry model meant to simulate a farm setting and varied the level of interaction between the two species [58]. When modeling a farm with both swine and poultry, the results of the model indicated that contact between animals increased infection in both, had higher numbers of infection, and the peak of infection was reached sooner [58]. Additionally, these changes were more noticeable in the swine population; this result is related to the parameterization differences between the species rather than an underlying characteristic of the model structure [58]. Zhuang et al. did not consider the epidemic dynamics for birth, death, and immigration/emigration parameters [58]. No interventions were considered in these models. These models were purely theoretical and indicate a need for further investigation of this multi-species dynamic.

#### 3.2.4. Swine–Human Models

Models considering both swine and human populations focused on investigating the effectiveness of control strategies and the impact of interspecies interaction on infections in both populations (Table 3). Two of the swine–human models were purely theoretical mathematical analysis and did not provide practical details about the scenarios or environments where humans and pigs are interacting. Additionally, the construction of these models led to uncertainty in the applicability of the results as model parameterizations are made for a generic setting. For example, Adi-Kusumo investigated infection dynamics between animals and humans [59]. The author implemented the simplest control method modeled by decreasing the interaction parameter value related to swine–human interaction; this control measure did not eliminate the disease transmission when the disease was endemic in the pig population [59]. The primary result of the Adi-Kusumo model is the endemicity of the animal virus impacting how endemic the disease will be in humans [59]. Widayanti et al. developed their model to investigate which parameters had the greatest impact on transmission dynamics between swine and humans. [62]. Vaccination was incorporated into the human population, but the study did not investigate how vaccination impacted the model outcomes despite vaccination rates being varied for the four simulation scenarios presented in the paper [62]. They showed that if the death rate parameters are lower than the pig mobility rate, the likelihood of susceptible pigs becoming infected, and the coefficient of periodic transmission, then the virus will become endemic in the pig population [62].

Kontowicz et al. developed a model to quantify the effectiveness of control measures targeting workforce and swine in a single facility [60]. Other strategies proposed for the control of the disease were having a directional workflow, vaccination, and isolating infected pigs [60]. Based on their simulations, a routine of working from the youngest batch of pigs to the oldest has the potential to reduce the risk of transmission in pigs [60]. In the cases where new strains emerge, the early identification and isolation of pigs can reduce the number of total infections and probability of infecting workers [60]. Vaccination was found helpful in reducing the total number of infected pigs and increased the time to the first worker infection, even when vaccines were between 20% and 60% effective [60]. One of the key limitations of this study was the potential underestimation of interspecies transmission and days to first workforce infection because they were calculated from a limited number of outbreak studies at outdoor agricultural fairs or research farms with a lower pig to worker ratio [60].

Dorjee et al. and Saenz et al. developed models with three linked sub-populations: swine, farmer worker, and general population [42,61] (Table 3). Saenz et al. investigated the dynamics of influenza in the community with a Concentrated Animal Feeding Operation [61]. The model demonstrated that the extent of the influenza epidemic in humans was amplified by 42–86% as the percentage of CAFO workers in the local community varied from 15% to 45% [61]. The amplification of the disease in the community surrounding the CAFO was canceled out when 50% of the workers were vaccinated and higher percentages of vaccination decreased the size of the human outbreak [61]. The transmission parameter for the human population was uncertain as it was calculated from R_0_ of historic pandemics of 1918 and 1957 and therefore could be an overestimation [61]. The work of Saenz et al. inspired the work of Dorjee et al. who altered the model to cover a larger geographical area [42,61]. Dorjee et al. developed a between-herds/households model for influenza transmission in Ontario, Canada, based on the NAADSM model. The goal of their analysis was to identify the transmission parameters that mostly impact disease transmission and investigate the impact of human vaccination on an epidemic. In this model, once the infection was introduced in the rural/urban populations, it would spread in these populations independently of its spread at the swine–human interface [42]. Regarding vaccination, targeted campaigns for farm workers that led to 60% coverage of that population reduced the epidemic size between 8 and 21% [42]. The simulation model uses farms and households as its units but cannot incorporate or explore the effects of different contact network structures [42]. Moreover, NAADSM can only assign a single location to each unit, limiting its flexibility in modeling multiple locations per unit [42].

Only one model in this category, Wong et al. [17], did not incorporate a control strategy. The objective of Wong et al. was to simulate an outbreak among county fair attendees to estimate the number of infections [17]. The model estimated the probability of human infection to be 0.024 for each minute in contact with the infectious swine; of the 14,910 individuals who attended the fair, the model estimated 80 (95% confidence interval [CI]: 40–133) individuals younger than 20 years and 56 (95% CI: 29–96) people who were 20 years or older who were infected with H3N2v [17]. The model was fitted to suspected cases data, and all demographic and contact behavior parameters were informed from a retrospective survey among agricultural club members who attended the fair [17]. The model did not consider different types of contact, such as observing pigs from a distance or directly handling the animals, which could vary among cohort members and other fair attendees. Moreover, the transmission dynamics of influenza among pigs were primarily informed by an R_0_ value from a previous modeling study using data from an animal experiment in isolation rooms.

## 4. Discussion

Mechanistic modeling of influenza in swine has been used to evaluate control strategies, investigate multi-species dynamics, and elucidate why the disease remains endemic on farms. Specifically, the farrow-to-finish models have identified piglets as a critical subpopulation in influenza endemicity on farms [46,47,48,51,52,60]. For example, they have shown that the constant influx of susceptible swine enables disease to persist in farrow-to-finish farm operations [46]. The results of the farrow-to-finish models are congruent with observations from surveillance studies conducted on farms. In a large-scale longitudinal surveillance study, weaned pigs between 4 and 12 weeks old have been identified as the most heavily infected subset of the population [63]. Similarly, in a cross-sectional study of four Brazilian farms with pigs of all age groups, the weaning population was shown to have the highest prevalence of infection [2]. One explanation proposed for disease being so high in this population is that these pigs are no longer receiving colostrum containing maternally derived antibodies (MDAs) [2,44]. There were two prevailing results for MDAs based on the modeling studies identified in this literature review. The results of Cador et al. indicate that MDAs are a contributing factor to endemicity as it delays the time until the infection dies out thereby increasing disease prevalence [46]. Conversely, Kontwicz et al. observed a 53% reduction in the number of simulations with an outbreak when MDAs were present [60]. Kontowicz et al. conducted an SEIR model with a human population whereas Cador et al.’s was an MSIR for swine only [46,60]. Contrary to Kontowicz et al., which focused its analysis on a single outbreak scenario by considering a fixed farm swine population, Cador et al. explicitly incorporated the swine population breeding cycle into their model [46,60]. The replenishment of susceptible hosts through the breeding cycle resulted in disease endemic behavior under suitable epidemiological conditions [46].

The question of whether to vaccinate swine herds is an integral part of swine husbandry and aims to minimize the economic consequences of influenza infection, especially in light of the 2009 H1N1 outbreak [21,26,44,53,64,65]. Toft et al. contradicts this view of vaccination by deeming it as too expensive as it lowered the profit margin [54]. However, it must be noted that Toft et al. is the oldest identified modeling paper and predates research quantifying reduced feed conversion ratios in infected fattening pigs [3,27,54]. After the 2009 H1N1 swine flu pandemic, the pork industry had a shift in perspective by becoming more willing to vaccinate their animals to regain consumer trust [26]. The focus of the modeling studies was to investigate if pig vaccination could eliminate influenza from swine farms, rather than evaluating if vaccination should be practiced at all [47,48,52,55]. Even though the models showed that vaccination does not eliminate influenza in these farm settings, it does reduce the transmission within the herd and spillover to humans [8,16,53,60]. In a transmission experiment by Romagosa et al., R_0_ was calculated for homogeneous and heterogeneous vaccines then compared with a naive population [53]. The naive swine population had an R_0_ value of 10.66 (95% CI: 6.57–16.46) while the R_0_ value of a swine population vaccinated with the homologous inactivated vaccine was 0 and the heterologous vaccine was 1 (95% CI: 0.39–2.09), indicating that vaccination could substantially reduce the risk of influenza transmission in swine farms [53].

In contrast to stated modeling goal swine vaccination, the goal of vaccination for the human population is to reduce disease prevalence among the population. Both Dorjee et al. and Saenz et al. showed that the vaccination of farm workers decreases the size of the human epidemic as it reduces the likelihood of disease transmission from pigs to humans [42,61]. While Saenz et al. considers a single community with a single facility, Dorjee et al. models a larger geographic area with multiple farm sites and fits the model to Ontario, Canada data [42,61]. A key difference in these models is their transmission unit, with Saenz et al. focusing on transmission between individuals and Dorjee et al. on transmission between households, despite generating similar conclusions about the community spread of influenza [42,61].

The transmission of influenza between swine and humans has been seen to occur at agricultural fairs during animal exhibitions [20,66]. In 2012, 10 out of 40 agricultural fairs in Ohio had swine tested positive for H3N2 with genomes a near match to the 320 cases confirmed in humans whose cases were traced back to the fairs [19]. Later in 2016, between July and August, seven fairs across Ohio and Michigan were associated with human cases [20]. Despite this increasing influenza zoonotic risk, only Wong et al. developed a model contextualized by this type of scenario [17]. A ban of swine exhibitions is not an economically feasible solution to mitigating the zoonotic risk of influenza in the USA. Based on 2018 economic data, the swine exhibition sector is worth an estimated $1.2 billion with the sales of pigs at agricultural fairs making up 33% of the industry net worth [67]. A greater than 3-year ban on swine exhibitions could lead to a 45% economic loss for the industry and a shift in species shown at the fairs [67]. Banning swine exhibitions is not the solution to ending zoonosis from agricultural fairs, as this may only shift the species spilling influenza into humans. More mathematical models need to be developed as a first step in evaluating control strategies to reduce the zoonotic transmission of the disease and to recreate past events to better quantify transmission risk to fairs attendees.

In this systematic review, the literature was limited to studies published in English only; therefore, the models published in other languages may have been missed. During this review, no models were identified for H1N2 and only one model for H3N2. Additionally, no models for influenza B and C were identified. Many of these models choose to use non-specific or a generic influenza A virus. The focus of most modeling studies has been on H1N1 because of the 2009 Swine flu pandemic. As a result, H1N2 and H3N2 viruses’ transmission are not being sufficiently modeled despite posing an imminent threat to both populations and swine because of bilateral transmission [19,20,68,69]. Currently, there are many gaps between research findings and the practical applications of the mechanistic models in domestic swine agricultural practices.

## 5. Conclusions

Mechanistic models are a useful method for analyzing the transmission of influenza in swine and investigating multispecies dynamics. These epidemiological models can also be used to design, evaluate, and identify optimal control measures to eliminate influenza transmission or mitigate its burden and zoonotic risk in swine production farms. There have been limited modeling studies related to the spread of H2N3 and H1N2 in farms, even though these two strains have been linked to spillover events to humans from infected swine [19,20,69]. To develop these models, additional research focused on the transmission of H1N2 and H3N2 in farm settings and the sharing of available case data is required, as models are only as good as the parameters used to generate their outputs. Considering that current control strategies are unable to eliminate influenza transmission from livestock herds and the potential for viral reassortment in swine, mechanistic models are paramount for designing and testing new biosecurity measures for preventing the next swine-related pandemic.

## Figures and Tables

**Table 1:** Search strings for identifying articles.

Category	Terms
Animal	Pig*, swine, porcine, suid
Disease	Influenza, influenza virus, flu
Modeling	mathematical model, Markov model, decision tree model, network model, decision tree analysis, metapopulation model, dynamic model, mathematical, mathematics, mathematic, model*, biomathematic, biomathematics, mathemati*, biomath, computational biology, computational, computational statisti*, mechanistic, equatio*, formul*, computer simulatio*, statistical mode*, statistical metho*, stochastic proces*, SIR, SEIR, susceptible-infected-recovered model, susceptible-exposed-infected-recovered model, compartmental, deterministic, stochastic, predictive mode*, predictive modeling, predictio*, epidemic theor*, discrete, continuous, applied math*, tempor* model, spat* model

*: Boolean operator for truncation and wildcard.

**Table 2:** Overview of Modeling Method.

Study	Model Structure	Transmission	Parameterization	Data	Model Fitting Method	Sensitivity/Uncertainty Analysis	Model Validation
Allerson, 2013 [44]	SIR model S: Susceptible I: Infected R: Recovered	Within herd	Model fitted to empirical data	Laboratory experiment conducted by authors	Generalized linear model using a log–log link function	None	None
Andraud, 2023 [45]	MSIVRS metapopulation model. M: Individual with MDA V: Vaccinated	Within herd	Model fitted to empirical data	Laboratory experiment conducted by authors	Bayesian inference performed with Monte Carlo Markov Chains. Maximum Likelihood	None	None
Cador, 2016 [46]	MSIR metapopulation model.	Within herd	Literature and assumptions	Simulated data	None	Probabilistic uncertainty analysis	None
Cador, 2017 [47]	MSIVRS model metapopulation model.	Within herd	Literature	Simulated data	None	None	None
Er, 2021 [4]	SIS model	Between herds	Model fitted to empirical data	Longitudinal seroprevalence data from 2009 to 2020	Bayesian inference of Bernoulli trials	None	Ground truthed to seroprevalence data from Norwegian herds
Etbaigha, 2018 [48]	SEIR metapopulation model. E: Exposed class	Within herd	Literature	Simulated data	None	Univariate	None
Mateus-Anzola, 2019 [49]	SIR model	Between herds	Literature	Simulated data	None	None	None
Nelson, 2015 [50]	SIR metapopulation model	Between countries	Model fitted to empirical data	Swine pop size, trade information from 146 countries from 1969 to 2010 reported to the Food and Agriculture Organization of the United States	None	Univariate	None
Pitzer, 2016 [51]	MSIRS model	Within herd	Literature and Model fitted to empirical data	Seroprevalence data from 15 Dutch finishing herds and 14 farrow-to-finish herds running from 290 to 4500 pigs per herd. H1N1, H1N2, and H3N2	None	Univariate	Ground truthed to seroprevalence data from Dutch herds
Reynolds, 2014 [52]	SEIR metapopulation model	Within herd	Literature	Simulated data	None	Univariate and probabilistic uncertainty analysis	None
Romagosa, 2011 [53]	SIR model	Within herd	Model fitted to empirical data	Laboratory experiment conducted by authors	None	None	None
Toft, 2005 [54]	SIR model	Within herd	Literature	Simulated data	None	None	None
White, 2017 [55]	SEIRV metapopulation model	Within herd	Literature	Simulated data	None	Latin hypercube sampling and partial rank correlation coefficient	None
Swine Avian							
Chen, 2016 [56]	Multispecies SIR model	Within herd	Literature	Simulated data	None	None	None
Coburn, 2011 [57]	SI-SIJ model J: super-strain infectious class	Within herd	Model fitted to empirical data and Literature	Human data calibrated to the 2004-2005 Thailand strain. Pig parameters were from the Food and Agriculture Organization of the United Nations	Least-squares	None	None
Zhuang, 2013 [58]	Multispecies SIR model	Within herd	Literature	Simulated data	None	None	None
Swine Human							
Adi-Kusumo, 2017 [59]	SEIR-SIRC model C: Cross-immune class	Within herd	Literature	Simulated data	None	None	None
Dorjee, 2016 [42]	SEIR-SEIR model	Between herds	Literature	Canadian population census and map data	None	None	None
Kontowicz, 2023 [60]	SEIR-SEIR model	Within herd	Literature	Simulated data	None	Probabilistic uncertainty analysis	None
Saenz, 2006 [61]	SIR-SIR model	Within herd	Literature	Simulated data	None	Univariate	None
Widayanti, 2019 [62]	SI-SIR model	Within herd	Literature	Simulated data	None	Univariate	None
Wong, 2013 [17]	SIR-SEIR model	Within herd	Model fitted to empirical data and Literature	Human infection cases and retrospective cohort investigation from interviews	Successive iterations of the differential equations model	Scenario Analysis	None

**Table 3:** General Epidemiology Information.

Study	Virus	Location	Model Type	Modeling Objective	Strategies Evaluated	Results	Limitations
Swine Only							
Allerson, 2013 [44]	H1N1 (IA/04)	Experiment	Stochastic compartmental	Estimate transmission parameters and the reproduction number in the presence of MDA	Vaccination (homologous vaccine: vaccine prepared with the isolate recovered from the specific population in which it will be used; heterologous vaccine: vaccine prepared with isolates distinct from the specific strain in the population)	No vaccination led to all 10 pigs becoming infected in 89% of the simulations Heterologous vaccination led to all pigs becoming infected in 80% of the simulations Homologous vaccination led to all pigs becoming infected in 0.3% of the simulations	Small herd size used in the stochastic simulations Pigs’ antibody titers were uniform and high, but this may not be the case for pigs in a production setting
Andraud, 2023 [45]	H1N1	Farrow-to-finish farm	Stochastic compartmental model	Evaluate single dose vaccination in weaned piglets when MDA	Vaccination at weaning age, mass vaccination, batch-to-batch vaccinations	The probability of the virus persisting ranged from 33 to 67% after 5 years post-introduction when sows were vaccinated leading to MDA in piglets No MDA in piglets with one vaccination led to the extinction of the virus periodically after 4 years post-introduction	The model considers dynamics of only one strain of influenza The results of the transmission experiment may not be accurate for transmission in a production setting
Cador, 2016 [46]	Influenza A	Farrow-to-finish farm	Stochastic compartmental model	Investigate the role of maternally driven immunity (MDA) on Influenza transmission	-	When greater than 66% of the piglets had MDAs, present model outputs were more variable The presence of MDA in piglets allows swine Influenza A to remain endemic in the population MDA lowers the piglet’s susceptibility thereby reducing the number of shedding animals at peak and longer epidemic durations Low airborne transmission did not decrease viral spread within the farm	No quantitative information was available to estimate the between-room airborne transmission rate For simplicity, authors focus on a single influenza strain
Cador, 2017 [47]	Influenza A	Farrow-to-finish farm	Stochastic compartmental model	Evaluate the risk of emergence of reassortment viruses from swine influenza viruses’ co-infection events	Vaccination, Concurrent export of weaning pig batches	The infection of growing pigs started at the farrowing site spreading to the nursery and finishing facilities. Three co-circulation patterns of subtypes were observed: (a) subtype *i* infection closely followed by subtype *j* (or vice versa), allowing brief co-infections; (b) simultaneous infections by both subtypes, resulting in moderate separate infections but many co-infections; (c) subtype *i* infection in young piglets, followed by subtype *j* in finishing rooms months later, causing two distinct outbreaks. Time between piglet batches impacts the fade out probability, large intervals favored fade-out. The gap between batches can prevent infection for older piglets to newly birthed ones Export of piglet batches was the most effective measure in facilitating fade-out of the virus, and there was a synergistic effect of disrupting infection dynamics through consecutive exports	No quantitative data was available for the quantity of virus shed by vaccinated animals. The model only included co-infection events because there was insufficient data to parameterize the likelihood of reassortant generation in the swine context
Er, 2021 [4]	H1N1 pmd09	Norway	Stochastic compartmental model	Determine if a Discrete-Time Markov Chain (DTMC) using yearly intervals can forecast influenza prevalence in Norwegian pig population	-	Good predictions of the 2010–2016 seroprevalence data when the model was fit using the 2009-2010 data. The data did not track well after 2016 because of changes in the force of infection	Considered an oversimplified model because it is an aggregated representation of all types in pig production Serosurveillance detects antibodies, so positive herds may not indicate new infections from an active virus. This is especially true for pig herds that tested positive in consecutive years
Etbaigha, 2018 [48]	Influenza A	Farrow-to-finish farm, Ontario, Canada	Deterministic compartmental model	Analyze the dynamics of influenza infection (including reinfection) in a farrow-to-finish swine farm and evaluate the effectiveness of control strategies	Vaccination, Reduction of Indirect contact	One infected gilt led to 50% of the gilt population being infected a few days later and oscillation is caused by weekly introduction of susceptible gilts No difference in infection dynamics was observed between growing pigs with and without immunity The vaccination strategies were unable to reduce flu burden experienced by entire farms Reducing indirect contact was also ineffective at reducing virus burden on the farms	No empirical data exist for parameters that impact the behavior of the model such as the vaccine immunity time and the indirect transmission rates For simplicity, authors focus on a single influenza strain.
Mateus-Anzola, 2019 [49]	Non-specific influenza	Backyard farms, Lerma, Mexico	Stochastic compartmental model	Investigate the impact of backyard farm networks on Influenza transmission dynamics	-	The connectedness of these farms is critical in understanding the dynamics of IAV In a low connectivity scenario, 34 days was sufficient to infect half the backyard farms In a high connectivity scenario, 5 days was sufficient to infect half the backyard farms.	Out of date national census information was utilized in the model Information on animal movement between backyard farms and markets at national, regional, and local levels was limited Information on pig density was limited
Nelson, 2015 [50]	Influenza A	Global trade network	Stochastic compartmental model	Investigate the global spread and swine influenza viruses and identify geographical hotspots for viral reassortment	-	The analysis shows continuous long-distance transmission of influenza along trade networks since the 1970s The United States and China are not representative of the observed virus diversity from the phylogenetic analysis and more surveillance efforts are required from Asian and Latin American countries	Did not account for within-country dynamics Did not consider the likelihood of initial viral emergence within seed countries The limited number of gene sequences from Asian countries other than China limited the ability to identify viral migration events within Asia
Pitzer, 2016 [51]	Influenza A	Farrow-to-finish farm, the Netherlands	Stochastic compartmental model	Investigate the effect of farm size and MDA on influenza persistence at the herd level Evaluate herd management strategies	All-in-all-out herd management	Virus persists in finishing herds with 1500 pigs when the R_0_ is 2, and in herds with at least 3000 pigs when R_0_ is 1.5 All-in-all-out herd management was the least likely to support IAV persistence If influenza strains are indeed capable of persisting at the individual farm level, their model suggests it may be difficult to detect through conventional surveillance efforts, since prevalence on the farm is predicted to be low	The model considers dynamics of only one strain of influenza Authors suggest that to have a 95% probability of detecting at least one positive pig for each strain at each time point, more than 45 pigs would need to be sampled randomly
Reynolds, 2014 [52]	H1N1 (IA04)	Experiment	Stochastic compartmental model	Assess the effectiveness of vaccination and demonstrate how partial protection impacts transmission dynamics	Vaccination (homologous and heterologous vaccine)	In the fully naive population, 84% of the simulations led to the entire pen becoming contact infected Heterologous vaccination simulations resulted in 7% having the entire pen becoming infected and 40% with no new cases Homologous pre-farrow vaccination delays the outbreak, lowers the number of infectious pigs at the peak by a third, and increases the number of infectious gilts and sows after the peak compared with no vaccination	Transmission rates could be overestimated due to the inoculation method leading to higher shedding of the virus Transmission parameters were calculated for within pen only and therefore cannot be applied to larger production scenarios
Romagosa, 2011 [53]	Non-specific influenza	Farrow-to-finish farm	Deterministic compartmental model	Analyze the dynamics of swine influenza infection dynamics at farm level and evaluate the effectiveness of vaccination strategies	Vaccination (homologous and heterologous vaccine)	IAV rapidly spreads after the introduction of one infected gilt through the breeding farm; at peak, more sows are infected than gilts in this section of the farm Breeding farm proportion of infected pigs was higher than the wean-finish farm Endemicity is predicted when influenza is introduced to a breeding farm Endemicity is not often observed in the wean-to-finish as no new susceptibles are added to the population Current vaccination strategies are not sufficient to eliminate IAV The sensitivity analysis showed the model was robust to changes in transmission rates	Limited model to a single influenza strain over limited timescale (40 days) Assumed fully naive population may have led to overestimation of infected pigs
Toft, 2005 [54]	Non-specific influenza	Fattening unit	Stochastic compartmental model	Create a framework to support decision making that optimizes control strategies for infectious diseases in pigs to maximize pig production	Vaccination, Treatment	Treatment was ineffective as it only reduced the contact rate by 40% compared with vaccination Vaccination is expensive and unprofitable to use in numerous scenarios assessed	Markov decision process models become large with complex decision problems Considered one section of animals despite production facilities having multiple sections that have the potential to infect each other Model assumes the number of infectious pigs is known and that there is no incubation period
White, 2017 [55]	H1N1	Farrow-to-finish farm	Stochastic compartmental model	To explore transmission dynamics of IAV under 14 different management interventions independently and in combination	Gilt introduction timing, Gilt separation, Gilt vaccination, Early weaning, Vaccination	Gilt introduction: lowered mean endemic prevalence compared to no control strategy, but the introduction of new gilts still caused an outbreak Gilt separations: minimal effects on endemic prevalence Gilt vaccination: minimally effective for disease control Early weaning: insignificant effect on max prevalence Vaccine strategy/efficacy: homologous mass vaccination every 2 months was the best Younger animals play a pivotal role in maintaining IAV in swine Overall vaccination only had a minimal effect	Did not evaluate different sized farms with all interventions Seasonality’s effects on transmission or introductions were not considered Did not account for differences in latency period and infectious period between piglets with and without MDA Model did not account for new strains being introduced, mutation of the virus, or partial immunity from past infections
Swine Avian							
Chen, 2016 [56]	Non-specific influenza	Purely theoretical	Deterministic compartmental model	Analyze influenza transmission between three species	-	Pigs as an intermediate host are more important than birds (the natural reservoir) in the role of human epidemics The per capita incidence rate from birds to pigs has minimal impact on the human epidemic	How species meet each other is not well defined Significant uncertainty in model parameters and results arises from calibration to generic settings
Coburn, 2011 [57]	Non-specific influenza	Thailand	Deterministic compartmental model	Analyze the dynamics of emergent influenza strain from recombination of avian and human strains	-	The emergence of super strains causes either: periodic outbreaks, a super strain that sustains at low levels without causing a big outbreak, or strong initial epidemic followed by weaker epidemics. Simulations showed recurrent outbreaks Super-strain emerging from pigs to humans usually had the highest mortality for humans at the first outbreak Cross-specie interaction makes the continuous introduction of super strains to humans inevitable	Limited data on influenza pigs meant authors had to make assumptions on parameters Model does not account for reassortment of the virus that could change transmission dynamics over the long term
Zhuang, 2013 [58]	Non-specific influenza	Farm with both swine and poultry	Stochastic compartmental model	Model multispecies influenza dynamics on a farm with swine and poultry	-	As the contact between animals increases, the infection in both populations reach their peak earlier and have higher values; this was more obvious in the pig population	Significant uncertainty in model parameters and results arises from calibration to generic settings Epidemic dynamics for birth, death, and immigration/emigration parameters were not considered
Swine Human							
Adi-Kusumo, 2017 [59]	Non-specific influenza	Purely theoretical	Deterministic compartmental model	Investigate infection dynamics between animals and humans	Reduced swine–human interaction	Higher risk of disease in the human population was observed when the virus was endemic in the swine population Viral persistence in the human population was shown to depend on the frequency of contact between humans and pigs	How swine and humans come into contact with each other is not well defined For simplicity, authors focus on a single influenza strain Significant uncertainty in model parameters and results arises from calibration to generic settings
Dorjee, 2016 [42]	pH1N1	Ontario, Canada	Agent-based model	Identify transmission parameters that impact the spread of disease most Investigate the vaccination’s impact on an epidemic	Human vaccination	It is critical to reduce influenza transmissibility at the swine–human interface as it prevents large outbreaks in the human population Targeted vaccination campaigns for swine workers were shown to reduce the proportion of swine worker households infected between 13%–68% Once the infection is introduced in the rural/urban human populations, the virus would spread independent of its spread at the swine–human interface	The unit of the simulation is farms and households Model could not incorporate or explore the effect of different contact network structures The personal computer version of North American Animal Disease Spread Model takes a significant amount of time to run With NAADSM, it is not possible to assign more than a single location to each unit
Kontowicz, 2023 [60]	Non-specific influenza	Commercial growing unit, Midwest United States	Stochastic compartmental model	Quantify the effectiveness of IAV control measures targeting workforce and hogs in a single facility	Vaccination, Isolation of infected pig, Alternative worker routine, Personal protective equipment (PPE) for workers	No interventions or maternal immunity led to 90% outbreaks for the simulations; this was reduced to 37.3% with MDA Vaccination is helpful, despite being suboptimal In the cases of new strains emerging, early identification and isolation of pigs can reduce the number of total infections and probability of infecting workers A routine of working from the youngest batch of pigs to the oldest may reduce the risk of transmission in pigs	Because of limited empirical data, the between-room transmission parameter combines both fomite and aerosol transmission The model may be using an underestimation of interspecies transmission and days to first workforce infection, because they were calculated from a limited number of outbreak studies at outdoor agricultural fairs or research farms with a lower pig to worker ratio
Saenz, 2006 [61]	Non-specific influenza	Concentrated Animal Feeding Operation	Deterministic compartmental model	Investigate the transmission dynamics of a new influenza strain among three linked populations (swine, farm workers, general population)	Human vaccination	The confined animal feeding operations (CAFO) workers serve as the bridging population for interspecies transmission, in the sense that only CAFO workers can infect and be infected by both humans and the CAFO species The extent of the influenza epidemic in humans was amplified by 42%–86% as the percentage of CAFO workers in the local community varied from 15% to 45% Higher successful vaccination levels lead to decreases in the size of the human epidemic. Vaccination of CAFO workers would be an effective use of a vaccine against a new influenza virus	Control measures were only considered for the worker population The basic reproductive number (R_0_) for humans was an educated guess based on large historical outbreaks
Widayanti, 2019 [62]	A-H1N1	Purely theoretical	Deterministic compartmental model	Understand transmission dynamics between pigs and humans	Human vaccination	If the parameters for death are smaller than the parameters for pig mobility, the chance of success of susceptible pigs becoming infected and coefficient of periodic transmission then the virus will become endemic in pig population	Significant uncertainty in model parameters and results arises from calibration to generic settings
Wong, 2013 [17]	H3N2v	County fair	Stochastic compartmental and Deterministic compartmental model	Simulation outbreak to estimate the number of infections among county fair attendees	-	Of the nearly 15,000 people in attendance, it is estimated 80 people younger than 20 years old contracted the infection and 58 people 20 years and older became infected Model estimates the probability of infection as 0.024 for each minute a person spent in contact with the infectious swine population at Fair A Low attack rate seen in this model; despite this, several infections can be expected from fair	Data used to fit the model came from suspected cases not confirmed cases The estimates of how long cohort members and other Fair A attendees spent in contact with swine may not be accurate The model did not account for different types of contact such as observing pigs at a distance or directly handling the animals, which could vary between cohort members and other fair attendees R_0_ was calculated from experimental studies and used to determine the number of infectious swine

## Data Availability

The search strings presented in this study are openly available in SearchRxiv https://www.cabidigitallibrary.org/journal/searchrxiv (1 August 2024): doi:10.1079/searchRxiv.2024.00588, doi:10.1079/searchRxiv.2024.00589, doi:10.1079/searchRxiv.2024.00590, doi:10.1079/searchRxiv.2024.00591, doi:10.1079/searchRxiv.2024.00592, and doi:10.1079/searchRxiv.2024.00593.
